# ASAS-NANP symposium: mathematical modeling in animal nutrition: overview of poultry nutrition modeling

**DOI:** 10.1093/jas/skaf140

**Published:** 2025-05-04

**Authors:** Edgar O Oviedo-Rondón

**Affiliations:** Prestage Department of Poultry Science, North Carolina State University, Raleigh, NC 27695, USA

**Keywords:** amino acid and energy metabolism, egg production, growth, nutrient deposition, poultry

## Abstract

Mathematical modeling has been used in poultry nutrition for the past 5 decades. Current amino acid recommendations for poultry have been based on mathematical models. This review aims to underscore the potential of modeling methodologies to minimize issues observed with the common use of empirical research, which researchers now realize. The review also discusses critical modeling issues and challenges to expanding modeling research. A comprehensive, although not exhaustive, list of existing models is presented to provide an overview of the efforts to develop these tools. Mechanistic models developed by EFG software and AVINESP are described in general terms since they have been well-documented over the past 3 decades. The framework and supporting data of these models are very similar. However, they differ in the research methodologies, including their parameterization and description of biological processes. The general methodology for model development and the fundamental equations are explained, and the current gaps in knowledge are discussed. The same concepts and description of growth, egg production, tissue and egg composition, and estimation of feed intake can be used to estimate needs for other nutrients and other animal species. The initial developments modeling poultry mineral nutrition are mentioned. Issues related to the accuracy and precision of these models might be resolved using big data, electronic sensors, portable devices to determine body composition, system-wide multi-omics, stable isotope technology, and machine learning techniques. Several publications have already demonstrated the practicality of integrating these methodologies. This review aims to demonstrate the relevance, applications, and solid basis of current mechanistic models that can be applied to advance sustainable poultry nutrition research. Modeling in poultry nutrition can help overcome many limitations observed using empirical methods and provide necessary decision-making tools. Models can be integrated with optimizers and feed formulation software.

## Teaser

This paper compares poultry nutrition modeling with empirical research. It describes the concepts, equations, and experiments used by 2 research groups to develop poultry mechanistic models and discusses how they can be improved with new technologies.

## Introduction

Over the past 45 yr, nutritional models have been increasingly used to determine the nutrient requirements of poultry (Harlow and Ivey, 1994; Oviedo-Rondón and Waldroup, 2002; Oviedo-Rondón, 2014; Leishman et al., 2023). This trend is evident in the past National Research Council Poultry Committees (NRC) reports. The NRC (1984) only had 4 references to mathematical models. In the 9^th^ edition, the latest published version, the NRC (1994), included over 300 references to mathematical models (NRC, 1994). However, the NRC committees for Poultry have tended to give more relevance to published peer-reviewed research designed to estimate energy and nutrient requirements based on empirical methods that rely on direct observation and measuring responses to nutrient levels in live experiments. Mathematical models were mentioned only as an alternative method due to the lack of empirical research on some specific nutrients. This limited acceptance of mathematical modeling within the scientific community stems partly from the fact that many scientists are more familiar with experimental research than systems thinking (Tedeschi, 2019). These developments remain in the early stages, as many researchers and professionals in the field still poorly understand the integration of experimental research data within mathematical modeling. Thus, the importance of modeling and its benefits for research and decision-making when nutritionists seek profitability have been minimized (Applegate and Angel, 2014; Oviedo-Rondón, 2014; Pesti and Choct, 2023). Models can also aid in estimating diets and feeding programs to minimize nitrogen and phosphorus excretion (Gous 2007, 2015a; Reis et al. 2023a) and can have a fundamental role in understanding the metabolism of nutrients and optimizing for profitability. This review paper describes poultry model development, highlighting its benefits, limitations, and the need for future advancements and integration with new technologies. Modeling can be helpful for sustainable poultry research and production.

## Empirical Research vs. Modeling in Poultry

Empirical research often utilizes dose–response, also called titration studies. These studies have been valuable for estimating the population’s requirements under specific conditions by quantifying the animal responses to dietary nutrient concentrations or intakes. However, despite the benefits obtained from empirical research, it is crucial to recognize its limitations. A primary issue is that the results may not always apply to other animal populations, feed intakes, growth rates, production levels, and environmental conditions (Gous, 1986, 2006). Continuous poultry genetic selection leads to variation in the genotypes used in the industry over time. Consequently, the nutritional data obtained with empirical research is rendered inaccurate. Moreover, the costs, time invested, and the limitations of animal use in research make empirical research unsustainable in the long run (Pesti and Choct, 2023; Klasing, 2024). Consequently, data obtained in animal research can gain more value when it is combined with mechanistic modeling.

The significant influence of the selected nutrient levels on each study also limits dose–response studies. These studies must include levels high enough to detect the maximum animal response and a level low enough to cause a deficiency. The responses to these nutrient levels can be modeled using linear and non-linear models. The selection of the model that fits the data best and the optimization method significantly influences the decision about the nutrient level that maximizes or minimizes the animal performance criterion (Kratzer and Littell, 2006). However, due to inconsistencies in the selection and optimization methodologies strategies among researchers, this factor causes variability in estimated values to optimize or maximize performance. The system thinking approach, backed by mathematical modeling, could assist researchers in creating better experimental designs and developing more robust recommendations for different production scenarios (Gous, 2006; Tedeschi, 2019).

Decision-making on nutrient dietary levels in the poultry and animal industry must consider economic constraints like the cost of inputs such as feed ingredients and the price of outputs or animal products to sell (Gous, 2015a ; Azevedo et al., 2021b). Under several market circumstances, maximum animal performance does not guarantee the best profitability for a poultry company (Chrystal, 2022; Pesti and Choct, 2023). In that decision-making process, the equations obtained in the empirical research to describe the animal responses to nutrient levels become more valuable than the optimum values estimated. However, most of the information in the equations generated is disregarded, and only single values of the optimum levels for animal performance are summarized in tables of nutrient recommendations. The description of growth and egg production responses of poultry to energy and amino acid (**AA**) levels have higher relevance for commercial applications and econometric analysis estimating the most profitable optimums (Talpaz et al., 1986, 2013; Roland et al., 1999; Ahmad and Roland, 2001, 2003a, b; Sohail et al., 2003; Gous, 2006; Reis et al., 2023b).

Empirical research has other limitations in making sustainable advances in poultry nutrition (Klasing, 2024). Each study tests only 1 or 2 nutrients or energy levels per experiment. Response surface designs like central composite (Fallah et al., 2020) and Bok-Behnken designs (De Leon et al., 2010; Liu et al., 2019; Maynard et al., 2021; Chrystal et al., 2022) can evaluate levels of 3 or 4 factors or nutrients. Integrating all the information on animal responses to multiple nutrients and their interactions is complex. Most researchers use univariate optimization to evaluate responses to single nutrient levels, but the complex interactions among nutrients have been overlooked.

This data analysis challenge can be solved by applying artificial neural networks with a multi-objective optimization algorithm. Mehri (2014) proposed this methodology to determine the optimum dietary levels of AA from the animal performance data obtained in experiments with a central composite design. Later, Mehri et al. (2016) applied this methodology to estimate the ideal profile of essential AA and metabolizable energy (**ME**) for growing Japanese quails. Morales-Suarez et al. (2021) also used this optimization concept to predict the nutritional requirements of essential AA in second-cycle brown-layer hens. However, the great majority of empirical research cannot wholly integrate information.

Generally, only lysine needs are determined in experiments, and all other AA are estimated based on ideal protein profiles. However, it is known that these ideal protein profiles may change depending on the animal’s performance or economic objective. Multiple ideal protein profiles have been proposed (Selle et al., 2023), and it has been suggested that nutritionists should move beyond the “ideal protein” concept to consider optimum ratios and specific amounts of all proteinogenic AA (Wu and Li, 2022). Wu and Li (2022) exposed 5 reasons to indicate that the ideal protein concept is imperfect in animal nutrition and may limit feed formulation. First, the composition of AAs in the diet differs from that in the whole body and varies with age. Second, essential AAs are degraded in extraintestinal tissues to resynthesize AAs. Third, the amounts and ratios of essential AAs are only part of the factors that affect animal responses. Fourth, the ideal protein concept does include the need for optimum metabolic processes and intestinal microbial metabolism. Lastly, the ideal protein concept ignores the needs of the small intestine and the reproductive and immune systems. This new concept can be easily incorporated into the existing poultry models.

The modeling equations used in previous NRC (1984, 1994) have been used mainly to determine only the quoted average optimum energy and AA requirements for multiple performance parameters under 1 specific condition. The equations or software used were never made available to the public, which would have allowed for dynamic utilization at various growth rates, production levels, and environmental conditions. The committee of the 10^th^ revised edition of the Nutrient Requirements of Poultry by the National Academies of Sciences, Engineering, and Medicine (**NASEM**) was expected to present a more mechanistic computer model. This software should incorporate mathematical equations that reflect the biological basis for predicting requirements and performance based on nutrient input-production response relationships. However, it seems that the econometric aspect still will not be included. In a preview of this NASEM poultry report (Klasing, 2024) presented on July 15, during the Poultry Science Association Meeting in Louisville, Kentucky, the members of this Committee indicated that a model would not be included or described in this edition. However, the committee still recommends developing mathematical models (Klasing, 2024). The main issue is that no research groups are currently dedicated to model development in the United States.

In contrast, over 24 yr, 4 updates to the Brazilian Tables for Poultry and Swine have been published since 2000. The 9th Edition of the Poultry NRC was published in 1994, and we are still waiting for the 10th edition (NASEM) to be published in 2025. The Brazilian Tables for Poultry and Swine (Rostagno et al., 2000, 2005, 2011, 2017, 2024) have been at the forefront of using equations to estimate energy and lysine requirements for broilers, layers, breeders, and quails since the first edition (Rostagno et al., 2000). The requirements were based on integrated factorial and empirical models in the recent release of the 5th edition of these Tables (Rostagno et al., 2024). Empirical models represent the animal response to nutrient levels or nutrient intakes observed in experiments rather than estimated by a model that seeks to explain the response based on physiological or metabolic knowledge that constitutes a theory. Setting these Brazilian tables apart are the detailed explanations of each prediction equation used to estimate energy or lysine needs to meet a target level of animal performance. This approach provides a comprehensive understanding of the calculation process. The effects of environmental temperature have been incorporated as quadratic functions into these prediction equations described in the Brazilian Tables (Morillo et al., 2023).

Using the factorial methodology, the nutrient levels needed for a target growth rate or performance level are determined by combining the estimated requirements for maintenance and production in the Brazilian Tables. These requirements are determined by describing the body weight, growth rate, proximate, and AA composition of tissues and the egg. This data is modeled with equations that are a function of body weight, metabolic body weight, or egg weight. However, the factorial methodology and most mechanistic models also have limitations. Only the average animal is modeled, and the population’s variability is assumed to be normally distributed, which may not be accurate for all growth and developmental traits. Then, the models may estimate nutrient levels that could be low for 50% of the population and too high for the other half. This issue can be overcome by simulating multiple growth or production rates and including the potential variability observed in the population (Gous and Berhe, 2006). Emmans and Fisher (1987) and Pomar et al. (2003) proposed generating a heterogeneous population of animals whose parameters were random, uncorrelated, and normally distributed, resulting in individuals with distinct requirements. Additionally, flock mortality can affect the results of nutrients and performance results for populations. This variability was then described in models using patterns of flock mortality (Grosskopf and Matthaus, 1990).

Factorial models set only as a function of body weight could be inaccurate since body composition and the deposition rate of chemical components may vary by genotypes, energy and nutrient intake, immunological challenges or disease, and environmental conditions. The solution has been to describe growth as the sum of the accretion of chemical components in the body (Sakomura 2004; Sakomura et al., 2005b). This approach is required if the goal is to maximize the efficiency of the nutritional program in a population of species in a production setting.

## Poultry Nutritional Model Evolution

The first model referenced in the NRC (1984) to estimate broilers’ energy and AA needs was developed by Hurwitz et al. (1978). Fisher (1983) and Hurwitz et al. (1983a, b) proposed models for estimating the AA requirements of turkeys. Models to estimate the energy requirements and AA of laying hens included the Reading Model (Fisher et al., 1973) and Hurwitz and Bornstein (1973). Since the early development of these models, multiple modeling approaches have been proposed by a few academic research groups and several private companies such as NOVUS International, Cargill, Aviagen, and Trouw Nutrition (Oviedo-Rondón, 2014). Table 1 lists the mathematical models published or made publicly available with poultry nutritional implications. Some models use a series of equations based on empirical research obtained with large datasets. In contrast, other models are mechanistic based on theory and research designed to estimate nutrient utilization and deposition parameters. Some of these models included an econometric component or module seeking to optimize profitability and minimize environmental impact rather than only maximize animal performance (Talpaz et al., 2013; Oviedo-Rondón, 2014; Leishman et al., 2023).

**Table 1.** Poultry nutritional models developed

Target	Model name	References
Broilers	FORTEL	Emmans, 1981, 1994; Emmans and Fisher, 1987
	CHICKOPT	Hurwitz et al., 1978, 1980; Talpaz et al., 1986, 1991
	IGM	Harlow and Ivey, 1994
	OmniPro II	Ivey and Harlow, 1991, 1992, 1994; Fancher, 1999.
		Pesti et al. (1986)
		Liebert et al. (2000)
		King (2001)
		Guevara (2004)
	EFG Broiler Model	Gous, 2006, 2007, 2012; Chrystal, 2022
	Aviagen (Alabama, USA) with LIDM Software from Israel	De Beer, 2009, De Beer, 2010; Talpaz et al., 2013
	Feed2Gain	Ivey and Harlow, 1991, 1992, 1994Mehr
	Panorama	Cargill Animal Nutrition (USA, Brazil)
		Nutreco (Canada)
		Danisco Animal Nutrition (UK)
	INAVI, CENTRAVI	Bignon et al., 2007; Mathiaud et al., 2013; Meda, 2011, 2014; Nugues et al., 2013.
	Broiler growth model, LAVINESP, UNESP, Brazil	Sakomura et al., 2015; Hauschild et al., 2015; Azevedo et al., 2021a,b; Reis et al., 2022; [Bibr CIT0142][Bibr CIT0143]; Reis et al., 2022.
Layers	Reading Model	Fisher et al., 1973,
		Hurwitz and Bornstein, 1977
	Economic Feeding and Management of Commercial Leghorns	Roland et al., 1999; Ahmad and Roland, 2001, 2003a, b; Sohail et al., 2003
	Egg production model, LAVINESP, UNESP, Brazil	Bonato et al., 2016; Alves et al., 2019; Neme et al., 2005 Sakomura et al., 2005, 2019; Da Nóbrega et al., 2022; Reis et al., 2023c.
Broiler breeders	EFG	Gous et al. 2015b
	Egg production model, LAVINESP, UNESP, Brazil	Sakomura et al., 2003; 2005a; Sakomura, 2004.
Turkeys		Hurwitz et al., 1983a, b.
		Emmans, 1989
		Rivera-Torres et al., 2011a, b, c
Japanese quail		Da Silva et al., 2019
Ostriches		Gous and Brand, 2008 ; Olivier et al., 2024

The application of mathematical and computer models has been limited by the lack of formal education in modeling or systems thinking (Tedeschi, 2019) in the poultry sector. The limited understanding of these models’ principles and solid scientific basis comes from the narrow visualization of the multitude of disaggregated scientific publications throughout several years without references to link them to particular model development. The lack of education on model development and utilization for practical poultry nutritionists has been one of the main issues that have limited its implementation, validation, evaluation, and further development (Oviedo-Rondón, 2014; Leishman et al., 2023; Pesti and Choct, 2023). Consequently, most poultry scientists have opted to conduct empirical research to obtain poultry response information to nutrients or dietary modifications since animal use is commonly approved, experimentation costs are lower than in other species, and the time frame to obtain data from poultry is faster than conducting modeling. But, all these conditions are not valid anymore (Klasing, 2024).

### The evolution of mechanistic models for poultry

Models based on empirical research have become outdated and underutilized (Oviedo-Rondón, 2014). Mechanistic models continue to be developed and can be used with the new genotypes while improving prediction accuracy. Most mechanistic models currently are deterministic, modeling the average bird in a population. The stochasticity is applied by simulating multiple times according to the potential distribution of the population or modifying the most relevant factors causing variability (Emmans and Fisher, 1986; Pomar et al., 2003). Stochasticity has been added to the EFG (EFG Software https://efgsoftware.net/) layer and broiler breeder models (Gous and Berhe, 2006; Johnston and Gous, 2007), and the laying hen AVINESP model (https://www.poultrymodel.com) using Monte Carlo simulation (Sakomura et al., 2019).

French researchers at INRA developed INAVI (Méda et al., 2015)  and CENTRAVI (Bignon et al., 2007). INAVI is a mechanistic broiler growth model that describes the utilization of ME intake partitioned for maintenance, physical activity, thermal balance, and growth. CENTRAVI includes dietary effects like energy and AA content, particle size, effective temperature (temperature, humidity, and airspeed), and flock stocking density (Bignon et al., 2007; Méda et al., 2014).

The EFG (Fisher, 2015) and the AVINESP (Hauschild et al., 2015) models share similar theoretical or conceptual aspects but differ in some methodologies for estimations and terminology. The EFG models for broilers, broiler breeders, turkeys, and swine were developed by Gerry Emmans from Scotland, Colin Fisher from the United Kingdom, and Rob Gous from South Africa. The AVINESP models were developed at the State University of Sao Paulo in Jaboticabal, Brazil, under the direction of Dr. Nilva K. Sakomura. The AVINESP models have been developed for several species: broilers, broiler breeders, pullets, laying hens, and quails. These 2 mechanistic models are based on the theory developed by Gerry Emmans and collaborators ( Emmans, 1981, 1989; 1994, 1997; Emmans and Fisher, 1986; Emmans and Oldham, 1988). They pointed out that the accurate mathematical description of the animal genotypes and their genetic growth potential is critical to determining energy and nutrient requirements in any animal species. In the same way, the accurate mathematical description of egg production is critical to determining nutrients according to the stage of egg production and egg mass.

### Description of growth

The most basic modeling approach has been to fit empirical body weight data as birds grow to a mathematical function, developing what is commonly called growth models. These semi-mechanistic growth models have a non-linear structure, sigmoid shape, and specific biologically meaningful parameters. In poultry science, Gompertz (1825), Logistic, Richards, and von Bertalanffy functions have been commonly used to model the growth patterns of birds (Darmani Kuhi et al., 2010; Narinç et al., 2017). These functions fit better data of animals that reach the mature state, and in most poultry species, this stage occurs after 15 wk of age. Using growth data of immature animals causes inaccuracy in parameter estimation.

A more mechanistic approach was taken later (Emmans, 1981, 1988, 1989) by considering growth as the summation of the body’s chemical components (protein, lipid, ash, and water) rather than just the total weight. These chemical components are dynamically deposited into the body and feathers throughout the growth period until the animal reaches maturity when the animal is at a state of equilibrium with a rate of change near zero. This mature weight is expressed as body protein weight. In birds, 2 components must be differentiated, the body and the feathers (Selle et al., 2023), due to the differences in AA and chemical composition (Table 2). The concept applies to broilers, broiler breeder pullets, hens (Gous and Nonis, 2010; Gous, 2015b), table-egg pullets and hens (Gous, 2015b), turkeys, ducks geese, ostriches (Olivier et al., 2024) and any other bird.

**Table 2.** Comparison of the ranked amino acid profiles of feathers and Pectoralis major in broiler chickens. Selle et al., 2023

	Feathers	Pectoralis major	
Amino acid	(g/16g N)	(g/16g N)	Index
Cysteine	7.92	1.2	6.60
Serine	10.85	3.22	3.37
Proline	11.65	3.72	3.13
Glycine	7.24	4.53	1.60
Valine	7.6	5.13	1.48
Phenylalanine	4.93	4.25	1.16
Threonine	5.13	4.45	1.15
Arginine	7.33	6.89	1.06
Leucine	8.34	8.01	1.04
Isoleucine	4.93	4.98	0.99
Tyrosine	3.16	3.81	0.83
Alanine	4.47	5.75	0.78
Glutamic acid	10.69	14.78	0.72
Aspartic acid	6.71	9.28	0.72
Lysine	2.06	8.98	0.23
Methionine	0.59	2.85	0.21
Histidine	0.79	4.28	0.18

Linear relationship between breast muscle and feather amino acid profiles: *r* = 0.215; *P* = 0.407.

Diverse mathematical functions can describe the path to reaching a mature protein state. However, the Gompertz function is the most suitable for poultry growth data. To fit this function, it is necessary to have data on birds that reach the mature stage without environmental and nutritional constraining factors. This is called potential protein weight (Pt) estimated with Equation 1. Otherwise, a second derivative of this function can be used. Not all mathematical formulas match the data precisely, and periods of over- and under-prediction of growth can be observed. The Gompertz function has only 3 parameters with biological meaning, and protein weight over time is defined as:


$$\begin{array}{l} Pt\;\left(kg\right)\;=\;Pm\;\times \;e-e\;(ln(-\ln(u_{0}))\text{–}\;(b\times t)) \end{array}$$ (1)

where Pt is protein weight at a time *t* (kg), Pm is mature body protein weight (kg), *u*_0_ is the degree of maturity at birth (Pt_0_/Pm), *b* is the rate of maturing (d^-1^), and *t* is the age (d).

The derivative of the previous equation can aid in estimating daily protein deposition (g/d) as follows:


$$\begin{array}{l} PD\;(g/d)\;=\;b\;\times \;Pt\;\times \;ln(Pm/Pt) \end{array}$$ (2)

Lipid, ash, and water growth can be described by allometric coefficients about protein growth (ln*Y* = ln *a* + *b* × ln*X*). Body chemical composition data have been obtained by the serial slaughter methodology, with the corresponding error that the same bird cannot be evaluated as growing or aging to reach maturity.

This information can also be obtained in vivo using dual-energy X-ray absorptiometry (**DEXA**) in the same birds. The DEXA results include total mass, lean (protein–water) mass, fat mass, and total bone mineral content, which, despite being correlated, do not represent exactly body weight, protein, moisture, lipid, and ash. The DEXA profile does not measure feather growth or feather chemical composition (Alves et al., 2019; Gonçalves et al., 2020). This issue can be partially overcome by combining data from comparative slaughter with chemical analysis and DEXA. The body chemical composition of poultry potentially can also be estimated with handheld devices like near-infrared spectroscopy, **NIRS** (Patel et al., 2021; He et al., 2023), and bioelectrical impedance (Puñal et al., 2024; Zuo et al. 2024).

Once the growth genotype has been described, energy, AA, ash, calcium, and phosphorus requirements for potential growth can be predicted by factorial models similar to the ones used by the Brazilian Tables (Rostagno et al., 2024). These factorial models can be more accurate when expressed as a function of chemical composition, especially about body protein content. There is evidence to suggest that the lipid-free dry matter (protein plus ash) is of constant composition and that the growth rate parameters (B) for each component are the same for a given genotype (Emmans, 1988; Gous et al., 1999, 2024). This means that the ash component of the carcass can be predicted directly from the protein content using the isometric relationship between them. The water and lipid weights, which are related to the lipid-free dry matter weight of the carcass by a simple power function, can be predicted from the protein weight under non-limiting conditions by allometry (Gous et al., 1999).

Each genetic line or genotype has different genetic growth parameters that must be periodically measured as genetic selection advances. This modeling methodology requires that appropriate descriptions of growth rates and carcass composition under non-limiting conditions are conducted. While some researchers have periodically done this work for broilers (Hancock et al., 1995; Gous et al., 1999, 2019a, b, 2024; Vargas et al., 2020a, b), much more attention to chemical carcass composition is necessary and could be estimated at farm level.

However, under commercial conditions, several constraints can limit this genetic potential growth. Models have included a description of temperature and relative humidity, daily light/dark cycles to describe lighting programs (Ferreira et al., 2019; Morillo et al., 2023), mortality rates, restricted feeding, and general growth constraints that can simulate the response to vaccines, infections, and other issues related to husbandry (De Freitas et al., 2023).

### Description of egg production

Egg production can be expressed as the weekly rate or percentage of flock egg production, cumulative egg production per hen, and egg weight or egg mass per week. Egg production rate, egg weight, and the average age at the onset of egg production have value in estimating energy and nutrient requirements. The egg-laying curve expressed as the percentage of lay per week in laying hens or broiler breeders is similar in shape to the cattle lactation curve. Therefore, functions for describing the shape of the bovine lactation curve have been adopted for fitting mathematical functions to empirical data or modeling egg production (Anang and Indrijani 2006; Johnston and Gous, 2006; Wolc et al., 2015; Otwinowska-Mindur et al., 2016 ).

The mathematical functions evaluated in the literature to fit egg production curves are described in Table 3. They all include parameters with a biological interpretation (Savegnago et al., 2012). Up-to-date models where the parameters mentioned above are included are sparse regarding egg weight patterns during the production period (Johnston and Gous, 2006). The gamma function is the most often applied model for the laying curve (Wood, 1967; McNally, 1971) because it is relatively simple to apply, easy to interpret, and best fitting to the data. In addition, 1 or 2 mathematical functions have been used to describe the egg production curve. However, logistic (Adams and Bell, 1980; Cason and Britton, 1988; Yang et al., 1989), exponential (Cason and Britton, 1988), polynomial functions (Lokhorst, 1996), segmented polynomials (Fialho and Ledur, 1997), and smoothed intersecting straight lines (Grossman et al., 2000) have also been tested to model egg production (Narinç et al., 2014).

**Table 3.** Summary of mathematical functions tested to describe egg production

Model name	Mathematical function	Reference
Logisitc	Y = (A) / (1 + B e − C x)	Adams and Bell, 1980; Cason and Britton, 1988
MMF	Y = (AB + C × D)/ (B + x D)	
Polynomial Fit of nth degree	Y = B0 + B_1×_ + B_2×2_ + B_3×3_ + B_4×4_	Bell and Adams, 1992
Segmented polynomials		Lokhorst, 1996
Rational Function	Y = (A + B x)/ (1 + Cx + Dx2)	
Sinusoidal	Y = A + B cos (C x + D)	
Quadratic	Y = B0 + B1x + B2 × 2	
Gompertz function	Y = A Exp[−Bexp(−Cx)]	Foster et al., 1987
Non-linear		Savegnago et al., 2011
Modification Compartmental Model	Y = A exp(−Bx)/1 + exp(−C(−x − D))	Yang et al., 1989
Delay model		Manetsch, 1976; Van Sickle, 1977 , Galeano-Vasco et al., 2013.

Y, Average weekly egg production in a particular period of recording; x, The week (of production) in which egg production was recorded; A, B, C, D, Model parameters as defined in a particular model; A, is an asymptote (a scale of parameter); B sets, the displacement along the x-axis (the rate of decrease in laying ability); C sets, the week (y scaling) the reciprocal indicator of the variation in sexual maturity; D, the mean age of sexual maturity; B0, B1, B2, B3, B4 are coefficients under quadratic and polynomial function, x = times (week), and e, is mathematical constant. The model parameters A, B, C, D, and E were derived using the least squares curve fit method. The adequacy of the models has been obtained by the coefficients of determination (R^2^), Root Mean Square Error (RMSE), Akaike’s Information Criterion (AIC), and Bayesian Information Criterion (BIC).

More than 1 mathematical function is often needed to describe the whole growth curve. For example, the Adams-Bell model uses a logistic function to describe the initial rise to peak and a negative linear function to mimic the decline in egg production post-peak (Adams and Bell, 1980). The Lokhorst model (Lokhorst, 1996) uses polynomial functions and has had the best fit for egg production curves of several genetic lines. The Morgqan Mercer Flodin and compartmental models and their modifications (Gavora et al., 1971, 1982; McMillan, 1981; McMillan et al., 1986) have shown the best fit for weekly egg production and egg weight (Sharifi et al., 2022).

The rate of lay is determined by the ovulation rate, which varies amongst individuals and over time. The ovulation rate, in turn, is established by an interaction between the rate of follicle maturation and the rhythmic release of luteinizing hormone. It, therefore, makes sense that a model designed to predict the egg production of a flock of hens should start by predicting the ovulation rate for each hen. Consequently, other approaches, like the Delay model, have been evaluated (Galeano-Vasco et al., 2013). The Delay model, structured by age, can be used to model egg production by considering eggs as the individuals in the system and rate change in the number of eggs produced during a period as the substates are affected by environmental factors or the relationship of supply/demand for resources in the system (Galeano-Vasco et al., 2013).

The cumulative egg production shows a smoother trajectory than the weekly egg production rate. Darmani Kuhi and France (2019) fitted monomolecular, logistic, Gompertz, Richards, and Morgan functions to egg production records of laying hens and broiler breeders. They concluded that Morgan and Richards’s equations provided satisfactory predictions of weekly egg yield at different egg production stages, from early to late production.

Predicting the mean age of the first egg is necessary for accurate flock egg production predictions. The Bristol-Reading model (Lewis et al., 2002) predicts this value based on the genotype and the lighting program applied during rearing. The model predicts the effects of the age of photostimulation on flock egg production variability, calculates the proportion of the flock capable of responding to photostimulation, and produces either a normal or a bimodal distribution of ages at the first egg.

Johnston and Gous (2006) took a more mechanistic approach. These authors discussed the details of ovulatory models, including variations of internal and external laying cycle length (usually 24 h) and lighting programs’ effects to predict ovulation rates accurately. In a flock of hens, the mean ovulation rate at the onset of lay will be determined by the distribution of ages at the first egg and the initial sequence lengths or individual ovulation rates. The subsequent egg production rate will be affected by the mean sequence length that varies by the genetic strain of hens. Sequence length rises initially until the peak of egg production and later declines at different rates between individuals. Zuidhof et al. (1999) created the Sequence Analyzer software that calculates the mean sequence length, the number of sequences, the prime sequence length observed near the peak of production, and the mean pause length of each bird that causes the decline in egg production. The ovulation rate and the rate of lay are identical unless internal ovulations occur or the production of double-yolked or soft-shelled eggs causes interruptions to egg sequences. These issues can also be predicted using quadratic-by-linear functions for internal ovulation, exponential functions for double-yolked eggs, and line-plus-exponential functions for soft shells. Sakomura et al. (2019) took a stochastic approach by applying Monte Carlo simulation. Standard errors were added to all parameters of the Johnston and Gous (2007) model for egg production rates and egg weight components of laying hens and broiler breeders using information published by Ferreira et al. (2016) and Bendezu et al. (2018).

Egg weight can also be estimated mechanistically by predicting yolk size according to hen age, which is mainly set by genetics and initial body weight (Johnston and Gous, 2003, 2006, 2007). The albumen weight can be calculated with allometric equations that are genetically dependent, and the summary of both components can affect eggshell weight. Adding yolk, albumen, and eggshells provides the estimated egg weight (Johnston and Gous, 2003, 2006). Dietary factors like fatty acid composition, AA content, and environmental and immunological stresses may affect the allometric relations between albumen and yolk. These factors remain to be studied to improve the accuracy of the model.

### Nutritional modeling

Once body growth, egg production, and their chemical components are mathematically described, this information can be used to estimate each condition’s energy, AA, and mineral needs. Models must also have accurate dietary energy and nutrient content inputs to match the observed conditions.

#### Estimating energy requirements.

Modelers have used different systems to estimate energy requirements. Emmans (1994) proposed the effective energy (**EE**) system, which is an alternative scale to net energy (**NE**) systems. The EE yielded to a monogastric animal can be estimated as EE (kJ/g) = 1.17ME−4.2CP−2.44, where ME (kJ/g) is measured at, or corrected to, zero N-retention and CP (g/g) is the crude protein (N × 6.25) content of the feed ingredient. This system was adopted in the EFG models.

**Figure 1.**
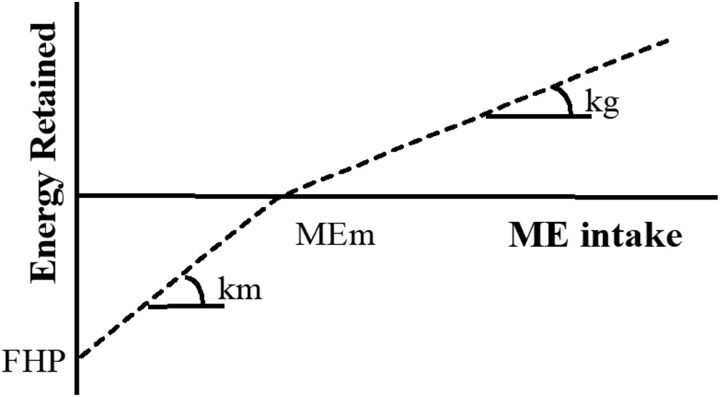
Relationship between energy retained and ME intake. FHP = fasting heat production; MEm = ME consumed for maintenance; kg = efficiency of energy utilization for growth; and km = efficiency of energy utilization for maintenance.

The AVINESP models (Table 1) use ME intake (**MEi**) and NE, which are partitioned to meet the maintenance, growth, and egg production requirements. In modeling, energy retention (**ER**) is represented by a 2-stage process (Fig. 1): the energy for maintenance (**MEm**) and the fasting heat production (**FHP**). The MEm is the energy intake when ER is zero. The FHP is the NE for maintenance. The slopes represent the partial energy utilization efficiency for maintenance (km) and growth (kg), with the MEi above maintenance increasing ER. A linear regression model is constructed from these assumptions: ER = MEm + kg × MEi. Considering MEm as the equation’s intercept and isolating the equation for (MEi), the following equation is obtained: MEi = MEm + (1/*a*) × (ER).

A more significant proportion of ER is assumed to be protein and lipid. However, the efficiency of energy deposition of lipids is higher than that of proteins. Therefore, the energy consumed above maintenance (**ER**) is retained as protein (**ERP**) and lipid (**ERL**), accounting for the differences in the efficiency of utilization. Therefore, the following equation is derived:


$$\begin{array}{l} MEi\;=\;MEm+(a)\times ERP+(b)\times ERL \end{array}$$

Where the reciprocal of the regression coefficients (1/*a* and 1/*b*) are the efficiencies of energy deposited as protein (*kp*) and lipid (*kl*). Shatnawi (2014) indicated that different values for *kp* (0.36 to 0.70) and *kl* (0.55 to 0.92) are found in the literature.

The models to estimate the energy required for egg production for broiler breeders and layers are described by this equation:


$$\begin{array}{l} MEi\;=\;a\times BW^{z}+b\times BWG+c\times EM \end{array}$$

where MEi is the ME intake, BW^*z*^ is the metabolic body weight, BWG is the body weight gain, and EM is the egg mass. The coefficients *a*, *b*, and *c* represent the maintenance, growth, and production requirements, respectively (Sakomura and Rostagno, 1993; Emmans, 1994; NRC, 1994).

The ME system may not account for the effect of diet on the energy efficiency of post-absorption nutrient metabolism. Consequently, the NE system has also been added in the models using similar concepts to partition the NE requirement in maintenance (**NEm**) and growth as protein (**NEp**) and lipid (**NEl**) deposition in the body or the egg (**NEe**):


$$\begin{array}{l} NE\;=\;NEm\;+\;NEp\;+\;NEl \end{array}$$

Studies have been conducted to obtain the MEm and NEm for laying hen pullets (Neme et al., 2005), broiler breeder pullets (Sakomura et al., 2003), broiler chickens (Sakomura et al., 2005a), broiler breeders (Rabello et al., 2006) and laying hens (Sakomura et al., 2005b), considering some of the factors that likely affect the requirement. In these studies, the comparative slaughter technique was used to determine the components of energy balance, i.e., ER, ERP, and ERL, and heat production (**HP**).

The MEm and NEm are affected by environmental temperature, degree of feathering, and genotype (Neme et al., 2005). These effects have been included in the AVINESP and EFG models. The degree of feathering affects the thermoneutral temperature, also called the critical temperature, which each bird will perceive. However, the degree of feathering is variable among individuals in flocks of breeders or hens and remains an issue to be investigated since current models only account for 0%, 50%, and 100% feathering. The MEm requirements were determined at thermoneutral temperatures as 112 kcal/kg BW^0.75^ for broilers, 92.4 kcal/kg BW^0.75^ for pullets, 144 kcal/kg BW^0.75^ for broiler breeder pullets, 113 kcal/kg BW^0.75^ for broiler breeder hens and 112 kcal/kg BW^0.75^ for laying hens. These values are used in equations that can be corrected by temperature.

The ME for gain (MEg) in laying hens and breeders was estimated to be 6.68 and 7.62 kcal/kg BW, respectively. There are differences in the efficiencies of depositing lipids and protein, which is more evident in pullets and broilers. On average, the ME needed to deposit a gram of lipid is 13.5 kcal/g, and for protein, 12.3 kcal/g (Sakomura et al., 2003; Longo et al., 2006). For egg production, the energy needed is 1.54 and 1.49 kcal/g for broiler breeders and laying hens (Rabello et al., 2006 ; Sakomura et al., 2005a.). The efficiency of ME utilization for egg production was 64% for broiler breeders and 62% for laying hens. Consequently, the ME requirement was estimated at 2.40 kcal/g of egg for both breeders and laying hens. Using all these coefficients, it was possible to determine models that determine EM requirements expressed in metabolic BW (kcal/kg BW^0.75^).

### Models to estimate requirements for amino acids for growing meat-type poultry.

For AA, the maintenance requirement has been generally defined as the digestible AA intake needed to maintain the nitrogen balance equal to zero. Studies to determine maintenance requirements for Lys, Met + Cys, Thr, Val, Trp, and Ile have been conducted using adult roosters (Bonato et al., 2011; Siqueira et al., 2013; Sakomura et al., 2015; Lima et al., 2016). But, alternative methodologies can be used in growing broilers (Dorigam et al., 2020). The AA maintenance requirements can be expressed in mg/kg BW as a function of metabolic BW (mg/kg BW^0.75^). Still, it is better to express them, avoiding the body’s lipid content, which varies depending on previous feeding (Gous et al., 2012). Reis et al. (2022) proposed equations to model the excess body lipid deposited when broilers are fed low crude protein diets. Another scale to express AA for maintenance is mg/kg of BP^0.73^, adjusted by the degree of maturity, which avoids the effect of the body’s lipid content (Emmans and Fisher, 1986).

The efficiency of AA utilization for body growth was determined using the linear regression slope between AA deposition and AA intake over maintenance. Following this concept, utilization efficiency (*k*) was 0.77 for Lys, 0.78 for Met + Cys, 0.73 for Thr, 0.73 for Val, 0.71 for Trp, and 0.69 for Ile (Reis et al., 2018).

These coefficients help to predict more accurately individual AA deposition in the body (AAD) and later AA intake needed in (mg/d) following equations: AAI = (AAm × BW^0.75^) + (AAD/k). The AAD = *a* + *b* × BWG. Then, the entire model will be equal to:


$$\begin{array}{l} AAI\;=\;\left(AAm\;\times \;BW^{0.75}\right)\;+\;\left[\left(a+b\times BWG\right)/k\right]. \end{array}$$

However, the feather growth should be expressed separately due to its specific AA composition and growth rate. Consequently, an alternative model proposed by Martin et al. (1994) for body and feather protein weight can also be used:


$$\begin{array}{llll} AAI\;=\; & [\;(AAm\;\times \;BP^{0.73}\;\times \;u)\; & \;+\;(FL\;\times \;FP\;\times \;AA\;f)\;+\;[\;(AAb\;\times \;BPD\;+\;AA\;f\;\; & \times \;FPD)/k\;]\;]\; \end{array}$$

where AAm is the AA maintenance requirement expressed as body protein (mg/BP^0.73^ m × *u*); *u* is the degree of maturity of the bird (*u* = BP/BPm); feather loss (**FL**) is expressed as a rate of loss, proportional to feather weight at 0.01 per day (Emmans, 1989); and FP is the feather protein weight in grams.

### The amino acid requirements for pullets and hens.

 Models have also been developed to estimate the AA requirements for breeder pullets and laying-type pullets reaching maturity (Silva et al., 2015b, 2021; Bonato et al., 2016). The models include protein accretion in reproductive organs, the AA pattern of the ovary and oviduct protein, and deposition efficiency in these organs.

The Reading model frequently estimates the AA requirements (Lys, Met + Cys, Thr, Val, and Trp) for egg production (Fisher et al., 1973). It was designed to determine the additional amount of AA worth feeding a laying hen population. This model considers that the response of each hen is described as an ascending straight line followed by a plateau, represented by a linear broken-line model. Since individuals differ concerning their body weight and breakpoint, i.e., their genetic potential output, the population’s response is curvilinear (Curnow, 1973). Considering this population variation to estimate AA intake, the model is classified as stochastic. The advantage of such a model is that it enables the calculation of each AA’s optimum economic intake based on the AA’s marginal cost and the marginal revenue for eggs. This amount depends on the variation in body weight and maximum (potential) egg output and the relationship between the marginal cost of the limiting AA and the marginal revenue for eggs. The model for a population is described as:


<tex−math>

where *a* and *b* are the coefficients for maintenance and egg output, respectively. The standard deviations of body weight and the maximum egg output are represented by σBW and σEO_max_, and Z represents the economic factor related to the ratio of marginal cost to marginal revenue.

The standard deviations of body weight and egg output are unique to each production system, and therefore, the values to be used as inputs in the model should represent the reality of each egg producer rather than generic values. Similarly, the marginal cost of AA in the diet and the marginal revenue for eggs are dynamic, changing frequently during an egg-laying period. However, based on the structure of the model described in this topic and knowing the productive performance of a flock and the market conditions, a nutritionist can elaborate feeding programs that ensure the optimum profit. Despite the practicality, the potential for further studies to determine maintenance and egg output coefficients and appropriate ratios with other AAs is vast. The Reading model has been used to estimate AA requirements for broiler breeders (Silva et al., 2015a), and it’s just the beginning of what we can achieve with this model.

Sakomura et al. (2019) described a mechanistic and stochastic approach to estimating energy and AA requirements of laying hens and broiler breeders. The process describes an accurate estimation of growth, egg production rate, and weights of egg components. These egg weight components are multiplied by 2.5 kJ and 0.21 g of ideal protein per g of yolk, 2.6 kJ and 0.13 g of ideal protein per g of albumen, and 1.2 kJ and 0.004 g of ideal protein per g of shell. The requirement for each AA is calculated from the proportion of nitrogen in the yolk (27 mg nitrogen/g) and albumen (17 mg nitrogen/g). As yolk deposition is a continuous process, it is assumed that 2 g of yolk is deposited each day. The efficiency of AA utilization from feed to egg protein was assumed to be 0.80 (Gous and Nonis, 2010). In this model, feed intake is an output of the model to indicate the amount of feed and nutrients to ingest daily to achieve a desired egg production and weight.

### Estimating feed intake.

 Once the energy and nutrient needs are estimated, predicting the voluntary feed intake under diverse conditions is important. The theory has indicated that birds eat to satisfy their energy requirements (Leeson et al., 1996). On the other hand, Emmans (1997) and Lewis and Emmans (2020)  have suggested that feed intake of animals can be predicted as a function of its value at maturity, with a quadratic function. However, these 2 theories may not always apply to broiler chickens that have been subjected to genetic selection for fast growth for decades. Modern broilers may ingest feed until they feel satiety. Consequently, the prediction of the maximum feed intake of broilers (Nascimento et al., 2020) and broiler breeders (Nascimento et al., 2021) has been made using physical properties of feed like the water-holding capacity (Taylor and Kyriazakis, 2021).

#### Modeling for other nutrients based on existing models.

 The existing models can be used to estimate other nutrients. Recently, a first model was proposed for calcium and phosphorus utilization and metabolism using the AVINESP model (Reis et al., 2023a). Gous (2024) proposed another model for calcium and phosphorus metabolism based on the information discussed by Salisbury et al. (2021) using the EFG broiler model. This mechanistic model can integrate real-time information on biomarkers of mineral metabolism.

### Mechanistic modeling and accuracy and precision

Despite the decades of evolution of mechanistic models and the solid biological and nutritional concepts used as theory for their design, deviations between observed data and predicted data are still observed in broilers (Harlow and Ivey, 1994; Oviedo Rondón et al., 2002a; 2002b, 2002c; Van Niekerk, 2004;  Indarsih and Pym, 2010; Oviedo-Rondón, 2015; Reis et al., 2023b), and layers (Reis et al., 2023c). The relatively small differences reflect the gaps in knowledge or quantification of some factor effects and the variability in the observed values. Model inadequacies point to biological considerations where data are insufficient to describe nature. However, the poultry industry requires more accuracy and precision in predictions.

To improve prediction accuracy, a blend between mechanistic models and using machine learning modeling techniques with big data, like neural networks, have been proposed (Roush, 2006; Ahmad, 2009; Ahmad and Golian, 2010; Faridi and Golian, 2011; Faridi et al., 2012). Neural networks have higher prediction accuracy for body weight in broilers (Roush et al., 2006b) and caloric and feed efficiency in turkeys (Mottaghitalab et al., 2010) than other equations like Gompertz. Neural networks can accurately predict production parameters in broiler breeders (Salle et al., 2003) and egg production in laying hens (Savegnago et al., 2011; Wang et al., 2012) and seek their economic optimization (Morales-Suárez et al., 2023).

#### New technologies and mechanistic modeling.

Leishman et al. (2023) explored the promising evolution of empirical and mechanistic models in poultry production systems and their dynamic interaction with digital tools and new technologies. Benchtop, portable, and inline NIRS, along with bioelectrical impedance (Puñal et al., 2024; Zuo et al., 2024) portable devices, are revolutionizing our ability to describe feed ingredient and balanced feed nutrient composition and energy values (Bastianelli et al., 2018) and body composition (Patel et al., 2021; He et al., 2023). The application of sensor-based technologies, the Internet of Things, computer vision, and sound analysis is reshaping our understanding of poultry housing conditions (Goyal et al., 2024). These advancements, coupled with precision feeding systems and unsupervised and supervised learning algorithms, promise to improve our ability to predict and manage animal responses significantly (Leishman et al., 2023).

Multi-omics technologies and system-wide multi-omics are crucial methodologies in understanding bird nutrient utilization (Ghafouri et al., 2021; Alagawany et al., 2022; Mao et al., 2023). These technologies are not only shedding light on gut health (Upadhyaya et al., 2019), feed additive effects (Brugaletta et al., 2022), and other aspects related to poultry health and productivity ( Goossens et al., 2022; Zampiga et al., 2018; Dehau et al., 2022), but also providing valuable insights into the biochemical pathways, metabolic rates, and nutrient utilization efficiency that still need to be better elucidated (Pelícia et al., 2018; Carter et al., 2019; Shehata et al., 2024). Integrating this information to understand the physiology and metabolic pathways can aid in improving mechanistic models that can enhance our ability to quantify animal responses and improve the accuracy of existing poultry models.

## Conclusions

Mathematical modeling has been part of poultry nutrition in the past 4 decades. Only the results of 1 optimization are published in tables of recommendations or guidelines, and only a few share the equations that can be used for other conditions. The application of their full capabilities has been limited due to a lack of education and understanding of all the solid scientific principles published as original research throughout the years. Many models have been discontinued due to low implementation and are unavailable on open access, limiting their use, validation, and improvement. Some models based on empirical research have lower prediction accuracy, mainly when predicting the results of new genotypes. The EFG and AVINESP mechanistic models were developed with a logical series of modules to predict ME, NE, AA, calcium, and phosphorus needs to meet growth and egg production targets. The equations that describe energy and nutrient utilization have been published and described. There are still gaps in the understanding of some processes and efficiencies. Modeling requires a periodic review of the description of potential genetic growth, egg production, and metabolic efficiency rates. Electronic sensor technology, big data analysis, machine learning, system-wide multi-omics, and stable isotope technologies can aid mechanistic models in improving their accuracy. There is a consensus that modeling is more sustainable for conducting poultry nutrition research and has become a powerful tool for poultry production optimization and minimizing nutrient excretion.

## Abbreviations

AA: amino acids
AAD: amino acid deposition
AAI: amino acid intake
DXA: dual-energy X-ray absorptiometry
EE: effective energy
ER: energy retention
ERP: energy retention as protein
ERL: energy retained as lipid
EO: egg output
FHP: fasting heat production
FL: feather loss
HP: heat production
NE: net energy
ME: metabolizable energy; Pm, mature body protein weight
Pt: protein weight
